# Improving Functional Capacities and Well-Being in Older Adults: Strategies in Physical Medicine and Rehabilitation

**DOI:** 10.7759/cureus.66254

**Published:** 2024-08-06

**Authors:** Amalia Vancea, Madalina Iliescu, Kamer-Ainur Aivaz, Marius N Popescu, Cristina Beiu, Luiza Spiru

**Affiliations:** 1 Physical Medicine and Rehabilitation - Ovidius Faculty of Medicine, Carol Davila University of Medicine and Pharmacy, Bucharest, ROU; 2 Physical Medicine and Rehabilitation - Ovidius Faculty of Medicine, Balneal and Rehabilitation Sanatorium of Techirghiol, Techirghiol, ROU; 3 Economic Sciences, Ovidius Faculty of Economic Science, Constanta, ROU; 4 Physical Medicine and Rehabilitation - Elias Emergency University Hospital, Carol Davila University of Medicine and Pharmacy, Bucharest, ROU; 5 Oncologic Dermatology - Elias Emergency University Hospital, Carol Davila University of Medicine and Pharmacy, Bucharest, ROU; 6 Geriatrics, Gerontology, Old Age Psychiatry and Longevity Medicine, Carol Davila University of Medicine and Pharmacy, Bucharest, ROU

**Keywords:** scale for identifying fall risk factors, functional independence measure, visual analogue scale, older adults, physical medicine and rehabilitation, well-being, quality of life

## Abstract

As life expectancy continues to increase, improving the quality of life (QoL) for older adults becomes an important issue. This study investigated the impact of a two-week intensive rehabilitation program at the Techirghiol Balneal and Rehabilitation Sanatorium on older adults’ QoL, focusing on physical and cognitive function. The study employed a comprehensive geriatric assessment to evaluate the progress of 156 patients over 65 from admission to discharge. We used the Scale for Identifying Fall Risk Factors (STRATIFY) scale to assess the risk of falling, the Visual Analogue Scale (VAS) to assess pain levels, and the Functional Independence Measure (FIM) to assess motor and cognitive abilities. The program included multi-parametric evaluations and personalized treatment plans. Statistical analysis of these data led to the following results: The STRATIFY scale showed a significant improvement in patient functionality and a decrease in the risk of falling during hospitalization, with a mean difference in scores between admission and discharge ranging from 0.141 to 0.372, with a p-value of less than 0.001, confirming the clinical significance of this improvement. The VAS showed a significant reduction in pain or symptom intensity, reflected by a mean decrease of -3.141 between admission and discharge. The FIM recorded a mean increase of 1.436 in patients' motor capacity between admission and discharge, reflecting improved adaptation to daily activities, especially in the areas of self-care, sphincter control, transfer, and locomotion. Social participation and health status were positively influenced, demonstrating the benefits of short-term, intensive rehabilitation. The two-week rehabilitation program significantly improved the QoL of older adult patients. These outcomes suggested that active aging strategies could be effectively integrated into medical and institutional care frameworks, highlighting the necessity for policies that support older adults’ involvement in economic and social contexts.

## Introduction

According to United Nations data, in 2022, there were approximately 727 million individuals aged 65 years or older worldwide, representing about 9.5% of the global population [1]. This number is increasing as more generations age and live longer. Projections suggest that by 2050, the number of older adults will exceed two billion, constituting nearly 20% of the world’s population [2]. This demographic shift poses substantial challenges for healthcare systems, which must adapt to meet the needs of an aging population.

Functional capacity and well-being are essential for maintaining the quality of life (QoL) in older adults. Functional capacity includes the ability to perform daily activities necessary for independent living, such as mobility, muscle strength, balance, and cognitive functions like memory and decision-making [3]. Cognitive function is closely linked to physical function; deterioration in one can accelerate decline in the other [4].

Well-being in older adults is influenced by several factors, including physical health, mental health, level of independence, social relationships, and living environment. Declines in functional capacity and cognitive function can significantly impact these aspects [5]. Sarcopenia, or the loss of muscle mass and strength, is associated with an increased risk of falls and greater dependency [3]. Cognitive decline can compromise the ability to perform physical activities and negatively affect QoL [4].

Given these realities, developing health, long-term care, and social protection systems to meet the needs of an aging population is a priority. The adoption of the UN Decade on Healthy Aging (2021-2030) led by the WHO emphasizes this urgency [6,7]. The COVID-19 pandemic highlighted existing gaps in policies and services for older adults, reinforcing the need for a global, focused approach to offer long and healthy lives with maintained autonomy and QoL [8,9]. 

Medical rehabilitation plays a fundamental role in restoring and maintaining health, closely linking rehabilitation and QoL. Rehabilitation services aim to restore and improve function and independence, leading to a better QoL by improving function, reducing pain and discomfort, recovering communication and social interaction abilities, providing psychological support, enhancing independence and social participation, and helping individuals adapt to changes and manage chronic conditions [10-12].

Despite the increasing focus on aging populations, there remains a gap in the literature regarding the efficacy of short-term, intensive rehabilitation programs tailored to older adults. Specifically, there is limited evidence on how such programs impact physical and cognitive functions in a relatively short period. This study aims to address this gap by investigating the effects of a two-week intensive rehabilitation program at the Techirghiol Balneal and Rehabilitation Sanatorium on the QoL of older adult patients. 

The primary objectives of this research are to assess the impact of the rehabilitation program on patients' physical functionality, as measured by the Scale for Identifying Fall Risk Factors (STRATIFY) [13], evaluate the reduction in pain levels using the Visual Analogue Scale (VAS) [14], and examine improvements in motor and cognitive abilities using the Functional Independence Measure (FIM) [15].

The hypotheses tested in this study are as follows: H1, which posits that patients' scores on the STRATIFY fall risk assessment scale vary significantly between admission and discharge; H2, which suggests that patients' scores on the VAS vary significantly between admission and discharge; and H3, which proposes that patients' scores on the FIM scale vary significantly between admission and discharge.

## Materials and methods

By testing the hypotheses presented in the introduction section, we aimed to offer a balanced and empirically grounded perspective on this issue, identifying strategies to maximize advantages and mitigate disadvantages.

The study was conducted through the following steps: 1) definition of the research problem; 2) development of the research objectives; 3) formulation of the research hypotheses, namely, patients' scores on the STRATIFY fall risk assessment scale vary significantly between admission and discharge (H1), patients' scores on the VAS vary significantly between admission and discharge (H2), and patients' scores on the FIM scale vary significantly between admission and discharge (H3); 4) conducting a literature review; 5) establishing the research methodology; 6) presentation of results; 7) discussion of findings; 8) addressing limitations and suggesting future research directions; and 9) drawing conclusions.

Research design

The study analyzed the efficacy of therapies applied to geriatric patients who underwent a comprehensive and personalized rehabilitation program. This program was based on the premise that patients were offered, to the maximum extent possible, all the services they needed in accordance with their state of health, within the limits of the existing infrastructure.

Patients were admitted with the goal of addressing, as far as possible, their entire medical problems, with the clear aim of improving their functional and cognitive parameters using all possible means available at the sanatorium level.

The research interest focused on the evaluation, in statistical terms, of such a program. It was a therapeutic evaluation study of a comprehensive program that was intended to be improved.

To assess the impact that the multidimensional rehabilitation process had on increasing the QoL of geriatric adults, we used a questionnaire in the present research, which we administered to a sample of 156 adults, aged 65 and over, who were hospitalized for a period of two weeks in the Techirghiol Balneal and Rehabilitation Sanatorium, during the 2020-2022 period.

Techirghiol Balneal and Rehabilitation Sanatorium, located in the picturesque town of Techirghiol in Constanța County, Romania, is known for the therapeutic use of the sapropelic mud extracted from Lake Techirghiol. This mud, renowned for its rich concentration of essential minerals and trace elements, is licensed exclusively to the sanatorium, and it has been thoroughly studied, proving to be effective in the relief and treatment of various health conditions, especially degenerative and inflammatory rheumatic diseases, as well as in post-traumatic recovery and in the treatment of neurological, gynecological, dermatological, and peripheral vascular conditions [16-18].

With a capacity to accommodate up to 1,000 patients and to provide around 6,000 medical procedures per day, the sanatorium stands out as the largest center of this type in Romania. It operates all year round, and it provides a wide spectrum of balneal-physical-kinetic treatments, focused on three therapeutic directions: recovery, curative treatment, and prevention. Through these services, the sanatorium meets the needs of patients with various conditions of the locomotor system, including degenerative, joint, inflammatory, and post-traumatic conditions, as well as neurological, gynecological, dermatological, and associated diseases, like respiratory, occupational, endocrine, and cardiovascular. This medical institution is an important pillar in the field of healthcare in Romania, contributing to the well-being and recovery of patients through a holistic and integrated approach to treatment [17,19].

After selection, only 300 patients qualified for admission for a two-week course of treatment. Based on the inclusion criteria, i.e., patients over 65 years old, diagnosed with vertebra-peripheral osteoarthritis, and without neurological sequelae causing impairments such as limb paresis, we were left with 156 patients. These hospitalized adults had more than two comorbidities, including vertebral-peripheral arthrosis, cardiovascular diseases, type II diabetes, neurological diseases, osteoporosis, thyroid gland disorders, obesity, and varying degrees of functional deficits.

The patient approach during the two-week treatment period was multidisciplinary, comprehensive, and holistic, offering personalized care tailored to the patient's needs at the time of admission and continuously assessing them throughout their hospital stay. To enhance well-being, mobility, and motor functionality and reduce pain, while also considering specific contraindications of therapies, such as the risk of cardiac decompensation, chronic venous insufficiency, the presence of osteosynthesis materials or implants, or certain skin disorders or sensitivities to procedures, patients underwent a suite of procedures. The types of procedures administered were categorized as follows: thermotherapy (applications or mud/paraffin baths), therapeutic massage, electrotherapy, kinesiotherapy, and hydro kinesiotherapy. Based on the treatment response, the attending physician adjusted the patient's treatment plan accordingly, introducing or excluding specific procedures.

The categories of services provided by the Techirghiol Balneal and Rehabilitations Sanatorium were as follows: a) balneary treatments (by using natural resources, such as mud and mineral water for therapy; these included saltwater baths, mud baths, cold and hot, wraps, paraffin, hydro-kinesiotherapy in salt water, heliotherapy, and herbal baths [20,21]); b) physiotherapy (laser treatments, ultrasounds, galvanic baths, magnetotherapy, ozone therapy, and electrotherapy, which benefited from the most advanced technology [20,21]); c) kinesiotherapy (individual or in a group, exercise and physical therapy programs adapted to individual needs, to improve mobility, strength, and coordination, using state-of-the-art devices for neuro-motor recovery, kinesiotherapy assisted by virtual reality, and occupational therapy assisted by medical robotics [20,21]); d) therapeutic massage (relaxation, lymphatic drainage, etc. with or without mud [20,21]); e) psychotherapy and counseling (psychiatric and psychological support and counseling for patients who need mental and emotional care [20,21]); f) nutrition program (dietary regimen adapted to medical needs and nutrition counseling to support the recovery process [20,21]); and g) recreation and relaxation activities to support general well-being [20,21].

According to the literature, a rehabilitation program requires a minimum of 10 sessions, carried out over at least 10 days, whereas the norm is two or three weeks with 10 to 21 sessions [22-24].

During the two weeks, patients received specialist medical consultations provided by physical medicine and rehabilitation doctors, as well as doctors specialized in cardiology, psychiatry, gynecology, or dermatology, among others, depending on the patient's needs, as well as initial, intermediate, and final evaluations of treatment results. Assessments were made during the clinical consultation provided by the attending physician, using numerous assessment scales and other specific parameters/indicators resulting from previous measurements/analyses or obtained in the sanatorium.

The program at Techirghiol, which followed the literature requirement for the duration of the program, usually lasted for two weeks, during which time patients underwent a minimum of five procedures per day and a minimum of 70 treatment sessions over 14 days. Under these circumstances, the validation of research dedicated to this type of intensive two-week program is both a medical and an economic challenge. Our study set out to investigate the effectiveness of such a program in terms of both functional and cognitive progress, clearly demonstrating through statistical validation that the program works.

The questionnaire developed by the authors for this research was based on the use of three scales: STRATIFY, VAS, and FIM. The scales were applied to the patients both at the time of their admission and at the time of their discharge, after the completion of the two-week treatment. The questionnaires were administered throughout the year 2022 at the Techirghiol Balneal and Rehabilitation Sanatorium.

The sanatorium provided a diversified palette of physical medicine and rehabilitation services, integrating, to a greater or lesser extent, all the categories of interventions that could be performed, benefiting from state-of-the-art equipment, well-prepared specialized staff, and an exceptional natural climate, bearing in mind the qualities of the mud and water from Lake Techirghiol.

Sample description

From a statistical point of view, the choice of the sample considered four key elements: effect size, statistical power, level of significance (alpha), and variation in the data. To ensure a statistical power of 80% (1-β = 0.80) to detect a significant difference, with a significance level of 0.05 (α = 0.05) and an estimated effect size of 0.5 (according to Cohen's criteria for medium effect sizes), G*Power analysis indicated a minimum required sample size of 54 participants for a t-test (difference between two dependent mean-matched pairs).

We used a convenience sample for this study. Inclusion criteria in the sample were as follows: patients over 65 years of age, without major functional deficits (requiring ambulatory chair), with vertebral-peripheral arthritic disease, with similar personal pathological history (hypertension, diabetes mellitus, osteoporosis, and hypothyroidism), and Sars-Cov 2 negative. Exclusion criteria from the sample were as follows: post-acute or permanent neurological disorders such as hemiparesis, paraparesis, or tetraparesis; neoplastic disorders in the history; post-traumatic disorders, like fractures, polytrauma, or post-interventional (post arthroplasty, ligamentoplasty, and meniscus lesions operated). Basically, only patients with chronic, compensated, and under old drug treatment were chosen.

The demographic and social structure of this sample, presented in Table 1, indicated a predominance of women, who constituted 66% (103 persons) compared to men with 34% (53 persons). Most people in the sample were aged between 70 and 79 (51.9%), followed by those under 69 (40.4%), and only a small proportion, 7.7%, were over 80. Almost all individuals were retired (96.8%), with only 3.2% being employed. In terms of religion, the vast majority were Orthodox (96.2%), while a small fraction were Muslim (3.8%).

**Table 1:** Demographic profile of the survey respondents. Yellow: moderate risk of falling, red: high risk of falling, green: minimal risk of falling

	N	%
Gender		
Male	53	34
Female	103	66
Age		
Under 69	63	40.4
70-79	81	51.9
Over 80	12	7.7
Employment		
Retired	151	96.8
Employed	5	3.2
Religion		
Muslim	6	3.8
Orthodox	150	96.2
Marital status		
Married	99	63.5
Unmarried	57	36.5
Residence		
Rural	29	18.6
Urban	127	81.4
Frailty Index		
Yellow	83	53.2
Red	1	.6
Green	72	46.2
Total	139	100

In terms of marital status, 63.5% were married and 36.5% were unmarried. There was a clear split between the residence of the participants, with a predominance of urban (81.4%, 127 people) compared to rural (18.6%, 29 people). In terms of the frailty index (FI), those with a yellow score represented 53.2% (83 people), followed by those with a green score (46.2%, 72 people) and only one individual with a red score (0.6%). The total number of people who were assessed for FI was 139, representing 100% of the sample for this indicator.

Measurement scales

To achieve the objectives of the research, we used the following measurement scales: the STRATIFY scale, VAS, and FIM scale. They are internationally recognized as established tools for assessing the QoL on three essential levels: the assessment of functionality by measuring the ability to perform daily activities and the fall risk, the assessment of pain, and the assessment of functional deficit.

The STRATIFY scale, adapted from [13], was a functionality assessment tool that measures a person's ability to perform basic activities of daily living, such as walking, eating, washing oneself, using the toilet, dressing oneself, bathing, and others. Although it is not a specific tool for measuring the QoL, it is an important indicator of a person's independence and autonomy in terms of meeting basic needs. This adapted scale provided essential information to the medical staff regarding the fall risk, impaired functions (hearing, vision, ability to urinate), ambulation, balance disorders, postural hypotension, medication, lower limb impairment issues, and ability to communicate. Depending on the scores obtained by the patients during the assessment carried out using this scale, which can range from 0 to 11, 11 items being analyzed (each item can score the value 0 or 1), the patient was placed in one of the following groups: 0-2 points for green, minimal risk (no assistance required); 3-6 points for yellow, moderate risk (re-evaluation during hospitalization; assistance during treatment); and ≥7 points for red, high risk of falling (reassessment during admission; assistance during treatment).

The classification of patients from the very beginning into one of the three groups (green, yellow, and red) allowed for quick identification by the entire medical staff of the patient's needs and the adaptation of their conduct throughout the hospitalization accordingly. In combination with other assessment tools, the STRATIFY scale contributed to a more complete understanding of the QoL of older people or people with disabilities.

The VAS [14] is a self-assessment tool used to measure the subjective intensity of pain or other specific symptoms. Although this scale was not a complete QoL assessment tool, there was a link between the VAS and QoL, especially if pain had a significant impact on general well-being. If a person experiences chronic pain or severe symptoms, it could affect their ability to enjoy life, perform daily activities, sleep, and maintain social relationships. The patient was asked to complete a visual scale, which started from 0 (feels no pain) and reached 10 (the most intense pain alleged by the patient during their life), indicating the level of pain they perceived from a subjective point of view. The assessment of the severity of pain, as a subjective sensation, provided valuable information to the attending physician regarding possible limitations in the treatment to be administered to the patient, as well as the degree of impact that the pain sensation had on their daily life.

The FIM scale [15] is an assessment tool used for measuring a person's motor functionality. It assesses motor and functional abilities in various daily activities and provides a score for the level of independence in performing these activities.

The FIM scale included 18 items grouped into the following categories: FIM _AI: Self-care (feeding, grooming, washing/bathing, dressing, using the toilet); FIM _CS: Sphincter control; FIM _T: Transfers (bed, chair, toilet, shower/bathtub, etc.); and FIM _LA: Locomotion/ambulation (walking, climbing/descending).

All these provided a picture of the patient's motor capacity, synthesized in the Total Score Motor (TSM) variable.

Two groups of items that measure cognitive ability were added to the above-mentioned, namely, Comun: Communication (understanding, expressing oneself) and Integ_Soc: Social integration (socialization, family, memory, social management).

By adding the motor score to the cognitive score, a general FIM score was obtained, which allowed the patient to be placed into one of the following groups, depending on the level of functional dependence: mild functional impairment: total score FIM 36-41; average functional impairment: total score FIM 16 /17 points-34/35 points; and severe functional impairment: total score FIM 6-15 points.

In the research sample, we identified 70 patients with mild functional deficits, 83 patients with moderate functional deficits, and three patients with severe functional deficits.

QoL, as we have already stated, was influenced by a wide range of factors. The FIM scale, as well as the other scales used in the research, provided us with information on an important part of this puzzle, primarily referring to physical and mental health. Used together, they were able to provide an initial assessment of the patients upon entering the rehabilitation program, as well as a final assessment, thus statistically confirming our research hypotheses, according to which multidimensional rehabilitation programs can contribute substantially and effectively to the increase in the level of physical and mental health and implicitly to the increase in QoL [18,20].

Statistical methods

The systematization of the data, the processing of the questionnaire, and the administration of statistical tests were carried out with the help of IBM SPSS Statistics, version 28.0 (released 2021, IBM Corp., Armonk, NY).

The main objective of the survey was to assess the progress registered by the patients after the administration of the medical rehabilitation treatment related to functional independence, fall risk, pain symptoms, degree of mobility, and socio-cognitive status.

To analyze the differences between the two levels, at admission and at discharge, for the same sample, we used the paired samples T-test. This was a statistical technique used to directly compare two sets of observations obtained under different conditions (before and after the administration of therapies) but on the same experimental unit (the same sample of patients). This method allowed us to assess the effectiveness of the applied treatments by comparing pre- and post-intervention measurements.

## Results

The results of our research were organized according to the initially established hypotheses. The statistical information in Table 2 provides a detailed description of the 11 items described in the measurement scale section, which represented the variables used in the research.

**Table 2:** Descriptive statistics: paired samples The scores of these variables were recorded at admission (I) and at discharge (E): STRATIFY: Scale for Identifying Fall Risk Factors; VAS: Visual Analogue Scale; FIM: Functional Independence Measure; FIM _AI: self-care; FIM _CS: sphincter control; FIM _T: transfers (bed, chair, toilet, shower/bathtub, etc.); FIM _LA: locomotion/ambulation; TSM: total score motor; Comun: communication; Integ_Soc: social integration; TSC: total score cognitive; TS: total score

			Mean		N		SD	
Pair 1	STRATIFY I	3.64		74		1.130		
	STRATIFY E	3.38		74		1.190		
Pair 2	VAS_I	6.67		156		1.321		
	VAS_E	3.53		156		1.474		
Pair 3	FIM_AI_I	5.81		156		.788		
	FIM _AI_E	6.29		156		.693		
Pair 4	FIM _CS_I	6.30		156		.584		
	FIM _CS_E	6.38		156		.583		
Pair 5	FIM _T_I	5.99		156		.803		
	FIM _T_E	6.24		156		.701		
Pair 6	FIM _LA_I	5.40		156		.777		
	FIM _LA_E	5.99		156		.842		
Pair 7	TSM_I	23.46		156		2.533		
	TSM_E	24.89		156		2.435		
Pair 8	Comun_I	6.30		156		.647		
	Comun_E	6.36		156		.601		
Pair 9	Integ_Soc_I	6.23		156		.699		
	Integ_Soc_I_A	6.24		156		.704		
Pair 10	TSC_I	12.53		156		1.277		
	TSC_E	12.60		156		1.237		
Pair 11	TS_ FIM _I	36.01		156		3.628		
	TS_ FIM _E	37.50		156		3.429		

The selection was carried out in accordance with the general objective of the research and with the research hypotheses: identification of changes/variations at the motor level, both in general and by segments (locomotion, self-care, sphincter control, and transfer); identification of changes/variations at the cognitive level, both in general and by segments (communication and social integration); and identification of the general effects of the administered treatment, which we captured through the variations of the total scores on the three scales: STRATIFY, VAS, and FIM.

Paired sample comparisons, shown in Table 3, suggested significant differences between the mean values obtained at admission and at discharge.

**Table 3:** Paired-sample tests The scores of these variables were recorded at admission (I) and at discharge (E): STRATIFY: Scale for Identifying Fall Risk Factors; VAS: Visual Analogue Scale; FIM: Functional Independence Measure; FIM _AI: self-care; FIM _CS: sphincter control; FIM _T: transfers (bed, chair, toilet, shower/bathtub, etc.); FIM _LA: locomotion/ambulation; TSM: total score motor; Comun: communication; Integ_Soc: social integration; TSC: total score cognitive; TS: total score

		Paired differences				t	df	Significance	
		Mean	SD	95% CI of the difference				One-sided p	Two-sided p
				LO	UP				
Pair 1	STRATIFY E- STRATIFY I	-0.257	0.498	0.141	0.372	4.433	73	<0.001>	<0.001>
Pair 2	VAS_E – VAS_I	-3.141	1.086	2.969	3.313	36.116	155	<0.001>	<0.001>
Pair 3	FIM_AI_E – FIM _AI_I	0.487	0.501	-0.566	-0.408	-12.135	155	<0.001>	<0.001>
Pair 4	FIM _CS_E- FIM _CS_I	0.077	0.267	-0.119	-0.035	-3.594	155	<0.001>	<0.001>
Pair 5	FIM _T_E- FIM _T_I	0.250	0.449	-0.321	-0.179	-6.954	155	<0.001>	<0.001>
Pair 6	FIM _LA_E- FIM _LA_I	0.583	0.544	-0.669	-0.497	-13.386	155	<0.001>	<0.001>
Pair 7	TSM_E – TSM_I	1.436	1.256	-1.634	-1.237	-14.284	155	<0.001>	<0.001>
Pair 8	Comun_E– Comun_I	0.058	0.234	-0.095	-0.021	-3.081	155	0.001	0.002
Pair 9	Integ_Soc_E - Integ_Soc_I_A	0.013	0.160	-0.038	0.013	-1.000	155	0.159	0.319
Pair 10	TSC_E- TSC_I	0.071	0.303	-0.118	-0.023	-2.907	155	0.002	0.004
Pair 11	TS_ FIM _E- TS_ FIM _I	1.487	1.317	-1.696	-1.279	-14.101	155	<0.001>	<0.001>

Table 4 illustrates the effect size for each pair of data analyzed, using two different calculation methods: Cohen's d and Hedges' correction. Effect size, measured by Cohen's d, is an indicator that reflects the difference between two mean values expressed in terms of standard deviations (SD). Therefore, a Cohen's d value of 0.2 indicates a small effect, 0.5 a medium effect, and a value of 0.8 or greater signifies a large effect. Hedges' correction is a Cohen's d-adjusted method that adjusts for bias in effect size estimation, and it is particularly useful for small sample bias. It incorporates a correction factor in order to provide a more accurate estimate of the effect size.

**Table 4:** Paired-sample effect sizes ^a^The denominator used in estimating the effect sizes. Cohen's d uses the sample standard deviation of the mean value difference. Hedges' correction uses the sample standard deviation of the mean difference, adjusted by a correction factor. The scores of these variables were recorded at admission (I) and at discharge (E): STRATIFY: Scale for Identifying Fall Risk Factors; VAS: Visual Analogue Scale; FIM: Functional Independence Measure; FIM _AI: self-care; FIM _CS: sphincter control; FIM _T: transfers (bed, chair, toilet, shower/bathtub, etc.); FIM _LA: locomotion/ambulation; TSM: total score motor; Comun: communication; Integ_Soc: social integration; TSC: total score cognitive; TS: total score

Paired-sample effect sizes						
			Standardizer^a^	Point estimate	95% confidence interval	
					Lower	Upper
Pair 1	STRATIFY E- STRATIFY I	Cohen's d	0.498	-0.515	-0.756	-0.271
		Hedges' correction	0.503	-0.510	-0.749	-0.268
Pair 2	VAS_E- VAS_I	Cohen's d	1.086	-2.892	-3.248	-2.533
		Hedges' correction	1.092	-2.878	-3.233	-2.520
Pair 3	FIM_AI_E FIM _AI_I	Cohen's d	0.501	0.972	0.780	1.161
		Hedges' correction	0.504	0.967	0.776	1.155
Pair 4	FIM _CS_E- FIM _CS_I	Cohen's d	0.267	0.288	0.127	0.447
		Hedges' correction	0.269	0.286	0.127	0.445
Pair 5	FIM _T_E- FIM _T_I	Cohen's d	0.449	0.557	0.387	0.725
		Hedges' correction	0.451	0.554	0.385	0.721
Pair 6	FIM _LA_E- FIM _LA_I	Cohen's d	0.544	1.072	0.874	1.268
		Hedges' correction	0.547	1.067	0.869	1.262
Pair 7	TSM_E- TSM_I	Cohen's d	1.256	1.144	0.940	1.344
		Hedges' correction	1.262	1.138	0.936	1.338
Pair 8	Comun_E- Comun_I	Cohen's d	0.234	0.247	0.087	0.406
		Hedges' correction	0.235	0.245	0.087	0.404
Pair 9	Integ_Soc_I_A- Integ_Soc_I	Cohen's d	0.160	0.080	-0.077	0.237
		Hedges' correction	0.161	0.080	-0.077	0.236
Pair 10	TSC_E – TSC_I	Cohen's d	0.303	0.233	0.073	0.391
		Hedges' correction	0.304	0.232	0.073	0.390
Pair 11	TS_ FIM _E- TS_ FIM _I	Cohen's d	1.317	1.129	0.927	1.329
		Hedges' correction	1.324	1.123	0.922	1.322

The results of the statistical tests in Table 4 have the following meanings: STRATIFY E - STRATIFY I: Here, we have a medium effect (-0.498 with Cohen's d and -0.503 with Hedges’ correction). Negative values suggest an improvement at the moment of discharge compared to admission. VAS_E - VAS_I: The effect is large (1.086 with Cohen's d and 1.092 with Hedges’ correction), indicating a significant reduction in pain intensity from admission to discharge. ​FIM_AI_E - FIM_AI_I: A medium effect is observed (0.501 with Cohen's d and 0.504 with Hedges’ correction), indicating an improvement in self-care. FIM _CS_E - FIM _CS_I: The effect is small (0.267 with Cohen's d and 0.269 with Hedges’ correction), suggesting an improvement in sphincter control. FIM _T_E - FIM _T_I: We have a medium effect (0.449 with Cohen's d and 0.451 with Hedges’ correction) for transfers, indicating progress in terms of transferability. FIM _LA_E - FIM _LA_I: Here, the effect is medium to large (0.544 with Cohen's d and 0.547 with Hedges’ correction), signifying an improvement in locomotion/ambulation. TSM_E - TSM_I: A very large effect (1.256 with Cohen's d and 1.262 with Hedges’ correction) indicates a significant improvement in motor function. Comun_E - Comun_I: The effect is small (0.234 with Cohen's d and 0.235 with Hedges’ correction), showing a modest improvement in communication. Integ_Soc_I_A - Integ_Soc_I: The effect is small (0.160 with Cohen's d and 0.161 with Hedges’ correction), suggesting a slight improvement in social integration. TSC_E - TSC_I: The effect is small (0.303 with Cohen's d and 0.304 with Hedges’ correction), indicating an improvement in cognitive symptom control. TS_ FIM _E - TS_ FIM I: It shows a very large effect (1.317 with Cohen's d and 1.324 with Hedges’ correction), suggesting a significant improvement in total motor function.

## Discussion

The purpose of the STRATIFY tool was to assess patients' functionality and fall risk by measuring their ability to perform basic activities of daily living. The mean value decrease of -0.257 between STRATIFY E (discharge) and STRATIFY I (admission) indicated that, on average, patients had higher scores at discharge compared to admission. This suggested an improvement in their functionality and a decrease in the risk of falling during hospitalization. We were reasonably confident that the true mean value difference in scores was between 0.141 and 0.372, indicating a consistent and statistically significant improvement. The p-value of less than 0.001 for both one-sided and two-sided tests confirmed that the observed difference was not due to chance, but rather represented a real and clinically significant improvement.

These findings were consistent with existing literature, which underscored the effectiveness of rehabilitation interventions in reducing fall risk in older adults. For example, the study conducted by Giovannini et al. [25] highlighted that fall risk in older adults can be significantly reduced through the use of multicomponent interventions. These interventions included careful clinical assessment, identification of risk factors, and implementation of personalized rehabilitation strategies, such as physical exercises aimed at improving muscle strength and balance.

In addition, our study aligned with the conclusions of the study conducted by Montero-Odasso et al. [26], which showed that most clinical practice guidelines for fall prevention recommend risk stratification using gait and balance assessment tools, multifactorial interventions, medication review, and environmental modification.

Moreover, our study brought an element of originality by integrating a personalized rehabilitation program that not only targeted improving mobility and muscle strength but also included health education and pain management components. We placed special emphasis on involving patients in their own recovery process, providing educational sessions on fall prevention, proper use of assistive devices, and adapting the home environment to minimize risks.

This study included a demographically diverse population, providing a broader perspective on how different groups of patients respond to rehabilitation interventions. This allowed us to identify the specific needs and particularities of subgroups, contributing to the development of better-adapted rehabilitation strategies.

Therefore, these results suggested that patients experienced a significant improvement in their ability to perform basic activities of daily living between the moment of their admission and their discharge from the hospital, which confirmed H1.

Regarding the VAS, the mean value decrease of -3.141 between VAS_E and VAS_I indicated a significant reduction in pain intensity or symptoms as measured by the VAS in the patients from the moment of their admission to discharge. This suggested an improvement in their well-being. The SD value of 1.086 reflected the variability in terms of symptom reduction between patients, which was relatively small, indicating consistent change between the patients. The mean value decrease in pain intensity or symptoms was between 2.969 and 3.313, which are quite close and indicated the precision of our estimation, and the very high t-test value of 36.116 indicated a highly significant difference between the VAS scores at admission and at discharge. The extremely small p-value <0.001 for both one-sided and two-sided tests confirmed that the observed difference was statistically significant and most likely clinically relevant. Thus, the results indicated a significant improvement in the condition of the patients in terms of pain or symptomatology measured by the VAS Scale, from the moment of their admission to the moment of their discharge. This may reflect the effectiveness of the treatment received during hospitalization or the overall recovery of the patient, which confirmed H2. It was a positive indicator of the management of the patient during hospitalization.

Following the data analysis, we observed that patients who underwent thermotherapy had better results at discharge on the VAS. The therapeutic benefits observed may stem from the sedative and muscle-relaxing properties of thermotherapy, which included treatments such as saline baths, mud wraps, or paraffin applications. These modalities are known to stimulate local circulation through a vasodilatory effect and provide additional pain relief. Our results were in accordance with those of the study conducted by Brosseau et al. [27] and Fareed et al. [28], which demonstrated the beneficial effects of thermotherapy in osteoarthritis.

The data analysis also highlighted that patients with the marital status "unmarried" at the time of their presentation to the clinic had better results on the VAS scale both at the time of admission and at the time of discharge, compared to "married" patients. It is possible that the degree of pain tolerance for patients who live alone at home, compared to those with partners, was higher because they must carry out daily activities related to personal and household maintenance on their own, despite pain complaints and chronic diseases with decompensation potential [29]. In other words, those who live alone felt pain as a subjective sensation less intensely than those who lived as a couple. 

The results were consistent with those of the study conducted by Wade et al., which investigated the relationship between marital status and emotional suffering in patients with chronic pain [29]. The study found that although marital status was not associated with immediate pain intensity, there was a strong association with emotional suffering. Interestingly, widowed subjects experienced significantly less frustration, fear, and anger compared to all other groups (married, divorced, separated, or single). The authors suggested that the experience of losing a partner may have led to the development of additional coping strategies, providing a certain "emotional inoculation" against future lifestyle threats [29].

Thus, our study also highlighted the importance of marital status in modulating emotional responses to pain, indicating that unmarried or widowed patients may have developed resilience mechanisms that allowed them to manage chronic pain more effectively. These findings can have important implications for clinical interventions, suggesting the need for personalized approaches that consider the social and marital context of patients to optimize treatment outcomes.

From the perspective of how the scores on the FIM scale evolved regarding the functional capacity of the patients, both globally and by motor-cognitive segments, we observed several key aspects.

There was a significant increase in the general FIM score at discharge, primarily due to the improvement in locomotor functionality (self-care, sphincter control, transfer, and locomotion). Patients demonstrated greater capability in performing daily living activities, with some even transitioning from average to mild functional impairment. The mean value of patients' motor ability improved by 1.436 from admission (TSM_I) to discharge (TSM_E).

Regarding cognitive progress (communicative ability and social integration), the influence of the treatment appeared to be less significant. This may be attributed to the relatively short treatment period of only two weeks, which may have been insufficient to produce substantial improvements in communication and relational skills.

Overall, these results indicated improvements from admission to discharge on all the measured scales, with effects ranging from small to very large. These improvements could be attributed to therapeutic and medical interventions in the sanatorium, as well as to the natural recovery process. Thus, the medical interpretation of these results suggested that the treatment or rehabilitation applied in the sanatorium was effective in terms of improving the patients' QoL, as measured by various aspects, such as general health status, pain intensity, and functional capacity. These improvements were statistically and most likely clinically significant, reflecting a real improvement in the patients' condition.

The importance of rehabilitation programs carried out in spas is extensively covered in the literature. Researchers have shown positive results in terms of the health status of patients admitted to this type of medical institution while improving the parameters of their QoL across different types of pathologies [30,31].

Our results were consistent with the study conducted by Soboleva and Kaladze, which evaluated the effects of sanatorium rehabilitation on cytokine profiles and QoL in patients with juvenile rheumatoid arthritis [32]. Their study demonstrated that sanatorium treatments led to a significant reduction in pro-inflammatory cytokine levels (TNF-α, IL-1) and an improvement in QoL parameters. Similarly, our study showed that personalized rehabilitation improved QoL, but we achieved these results in a shorter time frame of just two weeks, compared to the longer treatment duration in the Soboleva and Kaladze study.

In addition, our study aligned with the conclusions of the research conducted by Koele et al., which showed that a 15-week multidisciplinary rehabilitation program for patients with chronic and generalized musculoskeletal pain led to significant improvements in pain, daily activities, and social participation [33]. Unlike Koele et al.'s study, which was conducted over a longer period, our study demonstrated similar improvements in just two weeks, using a diversified and intensive approach.

Therefore, our study made a significant original contribution, achieving good results in reducing fall risk (STRATIFY) and on the FIM scale, as well as on the VAS scale, in just two weeks. This success was due to the use of a personalized approach that included various types of therapies: electrotherapy, massage, physiotherapy, and thermotherapy (where applicable). Our approach was aimed at reducing patient disability and increasing QoL and engagement in daily activities.

An important aspect of our study was the broader range of pathologies and patients with multiple comorbidities for which we obtained positive results on a large scale. This underscored the versatility and efficiency of our personalized interventions.

While our study recorded positive results consistent with the literature, we believe that the analysis could be enhanced by incorporating additional assessment instruments at the initial and final evaluations of patients. These instruments could include the Tinetti Scale for assessing static and dynamic balance during gait, the Hachinski Score for cognitive status, the Edmonton Frailty Scale for frailty condition, the Mini Nutritional Assessment (MNA) for nutritional status, and the Up & Go test for muscle strength. These factors, along with suboptimal nutritional status and heightened pain perception, can precipitate frailty syndrome [34,35]. This scenario predicts substantial future expenditures for both the individuals affected and society at large, emphasizing the necessity for a medical paradigm that embraces the tenets of 3P medicine: predictive, preventive, and personalized [36].

Consequently, the adoption of a preventive strategy is imperative to intercept the early indications of pre-frailty and to implement timely interventions across the lifespan [37-39]. Our research is in harmony with current scholarly pursuits, mirroring the intensified interest in uncovering strategies that promote an active, healthy lifestyle and longevity, while simultaneously reducing associated costs [40,41].

The research on geriatric patients revealed that, although there were noticeable improvements in motor functions and pain management, the cognitive advancement during the treatment was less significant. This outcome highlighted important questions that set the stage for further scientific exploration. We questioned whether the treatment period was long enough to make a significant impact on the cognitive functions of older adults. We also considered whether the current counseling and social activities were well-designed to enhance communication and comprehension. Furthermore, we explored what additional services could be incorporated to enrich the overall intervention strategy and hasten cognitive improvement in these patients.

The interventions applied in our study were deeply individualized, being rigorously tailored to the needs and medical history of each participant. This customized therapy allowed for a more effective and directly targeted approach to each patient's specific problems, thus increasing the likelihood of seeing improvements in a short time frame.

The study was designed as an initial exploration of the effects of an intensive, customized intervention in a controlled and carefully monitored environment. The results indicated that notable improvements in functionality and QoL can be achieved even in a short period when interventions are well targeted to the specific needs of patients.

We recommend exploring the possibility of introducing creative and therapeutic activities, such as Art-Therapy, gardening, age-appropriate physical exercise, and Serious Games designed for cognitive stimulation, as well as workshops on various personal development topics. Given the cultural-educational diversity and variety of interests of the sanatorium’s residents, an innovative program that integrates these opportunities could be particularly beneficial [42].

The lack of a control group could be considered a limitation of the study, potentially influencing the ability to establish clear causality between the treatment and observed outcomes. However, the exploratory nature of our study aimed to assess the feasibility of the personalized approach in a real rehabilitation setting. This provided a solid basis for a subsequent, larger study, including a control group, to consolidate and extend our initial findings.

It is essential to evaluate the long-term stability of the improvements made during treatment. This can be done by monitoring a cohort of patients after their discharge, utilizing telemedicine and SMART technologies to track the sustained impact of the treatment and to offer continuous support, even remotely. In addition, there should be a thorough investigation into the complexity of frailty syndrome, employing every medical and statistical tool available for assessment. An in-depth evaluation of cognitive functions, nutritional status, and psychological factors, like depression, should be prioritized. This will facilitate the development of specialized prevention and treatment protocols for this syndrome within balneotherapy sanatoriums.

## Conclusions

Upon examination of the collected data and associated medical interpretations, we can derive several insights into the rehabilitation treatments administered to geriatric patients. The efficacy of these treatments hinges on a holistic geriatric evaluation, which ensures a tangible enhancement in the physical and mental well-being of the patients, thereby facilitating active aging and elevating their QoL. Treatments that are personalized and delivered through an intensive protocol, leveraging cutting-edge technology and optimal climatic conditions, have yielded notable health benefits.

In conclusion, our research underscored the significance of attentively structuring and coordinating treatment facilities in terms of location, service offerings, availability of medical and specialized staff, equipment, and advanced technologies. This challenge directly reflected the capabilities and adaptability of institutional and medical management. Such infrastructure was essential to enhance patient functionality and address their needs effectively, with the objective of securing observable and enduring results in both the medium- and long-term framework.
